# Ocean Lagrangian Trajectories (OLTraj): Lagrangian analysis for non-expert users

**DOI:** 10.12688/openreseurope.14133.2

**Published:** 2021-11-15

**Authors:** Giorgio Dall'Olmo, Francesco Nencioli, Thomas Jackson, Robert J. W. Brewin, John A. Gittings, Dionysios E. Raitsos

**Affiliations:** 1 Plymouth Marine Laboratory, Plymouth, England, PL1 3DH, UK; 2 National Centre for Earth Observation, Plymouth, England, PL1 3DH, UK; 3 Collecte Localisation Satellites (CLS), Ramonville Saint-Agne, 31520, France; 4 Centre for Geography and Environmental Science, College of Life and Environmental Sciences, University of Exeter, Penryn, Cornwall, UK; 5 Department of Biology, National and Kapodistrian University of Athens, Athens, 157 72, Greece

**Keywords:** Lagrangian analysis, geostrophic currents, non-expert users, satellite data, ocean colour, in-situ measurements

## Abstract

Lagrangian analysis is becoming increasingly important to better understand the ocean's biological and biogeochemical cycles. Yet, biologists and chemists often lack the technical skills required to set up such analyses. Here, we present a new product of pre-computed ocean Lagrangian trajectories (OLTraj) targeting non-expert users, and demonstrate how to use it by means of worked examples. OLTraj is based on satellite-derived geostrophic currents, which allows one to directly compare it with other in-situ or satellite products. We anticipate that OLTraj will foster a new interest in Lagrangian applications in ocean biology and biogeochemistry.

## Introduction

To analyse how the properties of a moving fluid evolve as a function of time, we can proceed in two different ways. We can study the fluid as it flows in front of us (e.g., by using a moored set of instruments), which is known as an analysis in a Eulerian reference frame. Or we can imagine to be moving with a particular parcel of the fluid and study how the properties of this parcel evolve with time (e.g., by collecting measurements from a surface drifter): this is called an analysis in a Lagrangian frame of reference (or “Lagrangian analysis”). Thus, while variations observed within a Lagrangian reference frame are purely temporal (i.e. occurring within a given water parcel as it flows along its trajectory), those observed in a Eulerian reference frame are not, because they are also due to the variations between the different water parcels that flow through the fixed point of observation.

In the ocean, these two types of analyses can generate significantly different results, depending on the strength of the currents relative to the size of the geographic area under exam, the duration of the study, and the strength of the local spatial gradients of the property being investigated. When current speeds are slow with respect to the ratio of the spatial to the temporal scales of the study, and the horizontal gradients are weak (i.e. small variations between the area of observation and its surrounding), Eulerian and Lagrangian approaches are expected to deliver similar results. Conversely, when the speed of the water masses is comparable to or greater than the ratio of the spatial to temporal scales of the investigation, and the gradients are strong (i.e. large variations within the area of observation), results from Lagrangian and Eulerian analyses will, in principle, differ.

For example, given a surface water mass with a typical velocity *υ* = 5 cm/s ( Lumpkin & Johnson, 2013), and strong variations of a water property over horizontal scales of 100 km, one could ask the question "If I want to analyse the evolution of the properties of this water mass over a temporal scale *τ* = 1 month, should I be considering a Lagrangian approach?". Since during such a study the water mass would move by a distance *σ* = *υ/τ* ≈ 130 km (which is larger than the spatial scale of the horizontal gradients), the above reasoning will lead us to conclude that a Lagrangian approach would be appropriate. Indeed, with a Eulerian approach, our observations would also include the variations induced by the advection of water masses with different properties from the original one. In more energetic regions, with *υ* = 25 cm/s, the distance traveled by the water parcel would increase to > 650 km, and hence a Lagrangian approach would be required even in presence of weaker horizontal gradients. Naturally, this rule of thumb should be used as a very first-order indicator to decide the reference frame in which the analysis should be implemented.

Although Lagrangian analyses have been traditionally applied by physical oceanographers to investigate ocean circulation and tracer dispersion (e.g., Blanke *et al.*, 2001; Döös, 1995), in recent years they have also emerged as an important tools for biological and biogeochemical studies. Lagrangian analyses can be used to determine the source and fate of a water mass sampled from a ship or by an autonomous platform, thus providing a dynamic framework for interpreting in-situ data and optimising field campaigns (e.g., d'Ovidio *et al.*, 2015) or to follow the evolution of biogeochemical properties detected in satellite data while separating changes due to advection from those due to biological processes ( Jönsson *et al.*, 2009; Lehahn *et al.*, 2011). Furthermore, Lagrangian analyses can be used to infer how spatially-separated components of a basin are connected by currents and how this connectivity impacts the life cycles of crucial ecosystem components ( Falcini *et al.*, 2020; Raitsos *et al.*, 2017). These are only some examples of a vast array of applications. However, so far, Lagrangian analyses have been restricted to “expert users”, who can convert velocity fields into Lagrangian trajectories.

Here, we present a new product of pre-computed ocean Lagrangian trajectories (OLTraj) that should allow non-expert users to more easily implement Lagrangian analyses. Below we describe how OLTraj was derived and we demonstrate how to use it by providing three practical examples.

## Data and methods

### Input velocities

The Ocean Lagrangian Trajectory product (OLTraj) was computed using reprocessed global daily multi-mission altimeter-based surface geostrophic velocities obtained from the Copernicus Marine Services archive from January 1998 to December 2019, gridded at 0.25° × 0.25° resolution ( Copernicus). This period was selected to overlap with the years of the available satellite ocean-colour observations.

### Calculation of trajectories

OLTraj was generated using the LAgrangian Manifolds and Trajectories Analyser (LAMTA), originally developed by d’Ovidio *et al.* (2004) and subsequently described in van Sebille *et al.* (2018). See section Software availability. The LAMTA code has been previously applied in support of in-situ Lagrangian experiments ( d'Ovidio *et al.*, 2015; Nencioli *et al.*, 2011) as well as satellite-based studies (e.g., to track Agulhas rings, Nencioli *et al.*, 2018). In OLTraj, trajectories are computed using a fourth-order Runge-Kutta scheme with an integration time step of six hours. During the integration, the geostrophic velocity fields are bi-linearly interpolated in space and linearly interpolated in time. For each day, we computed trajectories at a resolution of 1/8th of a degree in both latitude and longitude. Each trajectory extended backward and forward in time for 29 days.

### How Lagrangian trajectories are stored

The OLTraj product is stored in daily NetCDF-4 files. Each file contains the variables trajlat and trajlon that are the coordinates of the Lagrangian trajectories (latitude and longitude, respectively). trajlat and trajlon have three dimensions: lat, lon and time. time is of length 59, and its central time element ( time(30), hereafter *t*_0_) is the time from which the Lagrangian trajectories are computed forward (from *t*_0_ to *t*_0_ + 29 days) and backward (from *t*_0_ to *t*_0_ − 29 days). *t*_0_ corresponds to the time reported in the file name. lat and lon are the starting locations of the trajectories at *t*_0_ (before backward or forward advection). Hence, at *t*_0_ trajlat=lat and trajlon=lon. Elements 1 to 29 along the time dimension of trajlat and trajlon contain the coordinates of the backward trajectory. Thus, trajlat at time(29) contains the latitude values of the trajectory at *t*_0_ − 1 day, at time(28) that at *t*_0_ − 2 days, and so on until time(1) corresponding to *t*_0_ − 29 days, i.e., the last day of the backward trajectory. In the same way, the latitude values of the forward trajectory are stored in the trajlat elements 31 to 59. Similarly, the longitude values of the trajectory are stored in trajlon.

### How to access the OLTraj product

The OLTraj files were deposited as version 2.2 in open-access format at the UK Centre for Environmental Data Analysis (CEDA) archive. All files can be freely downloaded, but due to their relatively large sizes (~850 Mb each), we recommend accessing and subsetting them using the provided THREDDS data server (see README file).

### Examples

To demonstrate how to use the OLTraj product, we provide three practical examples as jupyter notebooks. These require the user to clone a publicly available repository and follow the instructions provided in the README file (see Software availability). The link to the Binder Launcher for each example is added next to the titles of the following sections.

**Figure 1.**
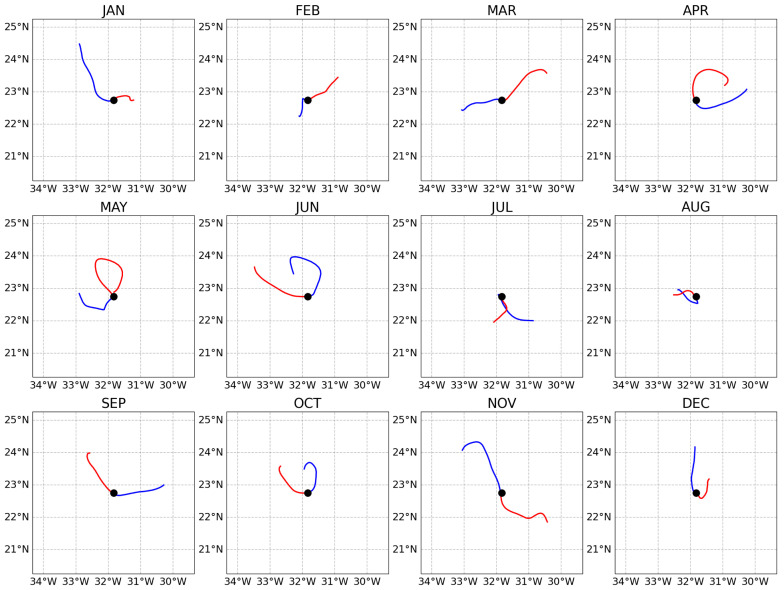
Backward (blue lines) and forward (red lines) trajectories of the water masses sampled at the fixed-point Bermuda Atlantic Time Series (BATS) station (black circles) during different months of year 2018 (presented in the different subplots). Each trajectory extends for 29 days backward and 29 days forward. The trajectories were extracted from the central day of each month.

***Example 1: Plotting trajectories around a fixed-point station ( Binder Launcher)***. The objective of this first example is to extract and plot the surface trajectories of the water masses sampled at a fixed-point station (e.g., the Bermuda Atlantic Time Series). These trajectories can be used to better understand the origin of the water masses sampled at the station and hence better interpret the variability observed in the time series. The coordinates of the station are used to extract OLTraj trajectories during each month of the year. Figure 1 presents the result of this example showing how surface water masses move differently during the year.

**Figure 2.**
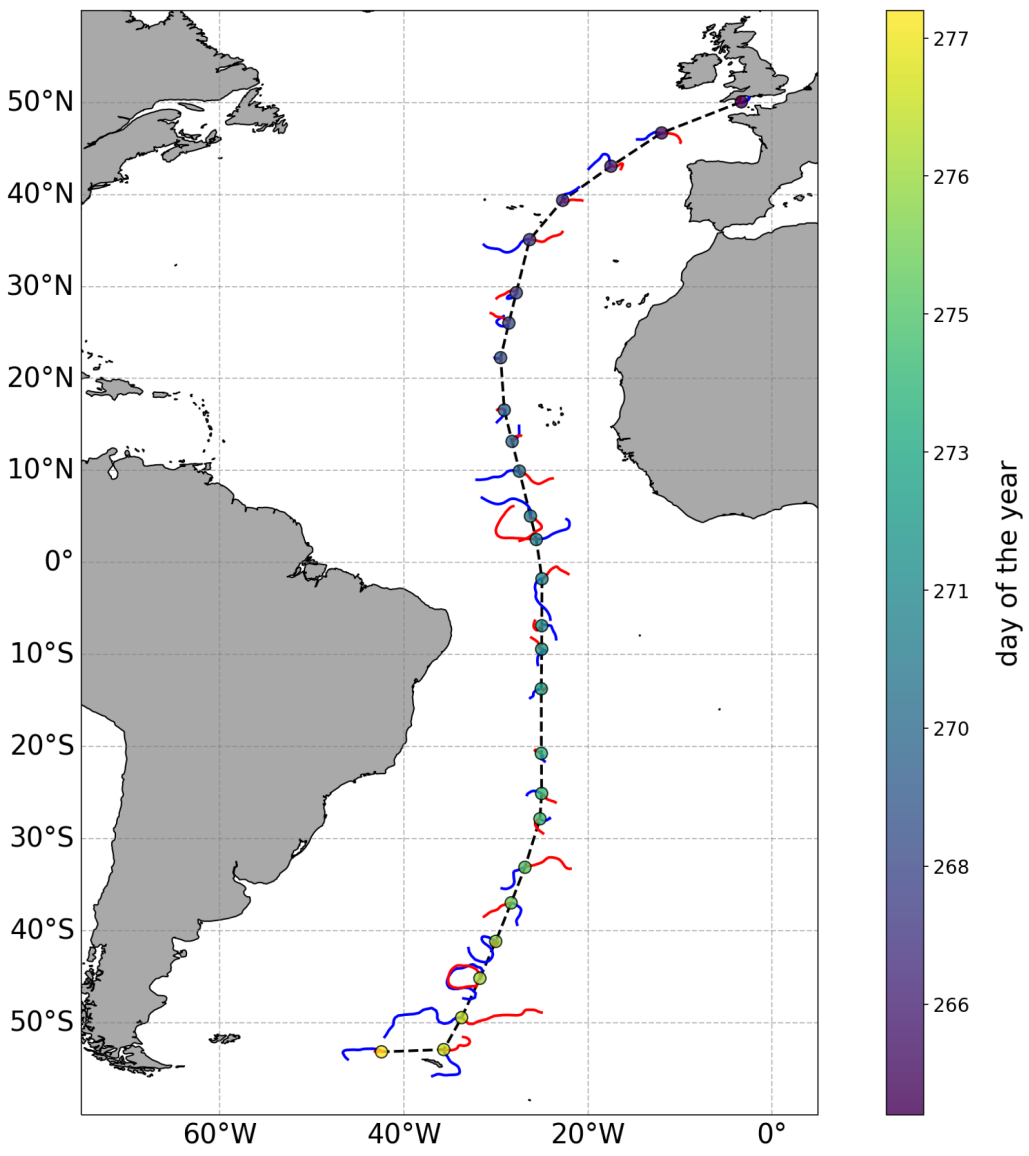
Backward (blue lines) and forward (red lines) trajectories of the water masses sampled at selected stations (circles, colour indicates the day of the year of each station) along the track (black dashed line) of a research expedition. Each trajectory extends for 29 days. Longer trajectories indicate that the sampled water masses were moving faster than those with shorter trajectories.

***Example 2: Extracting and plotting backward and forward trajectories along a cruise track ( Binder Launcher)***. The objective of this example is to plot the surface trajectories of the water masses sampled during a hypothetical research expedition. This information is particularly important because it can be used to better interpret the station-to-station variability measured during the expedition. The time and coordinates of the stations sampled during the expeditions are read from a text file and are then used to read and subset the corresponding OLTraj trajectories. Figure 2 presents the result of this example showing how different water masses along the expedition moved before and after they were sampled.

**Figure 3.**
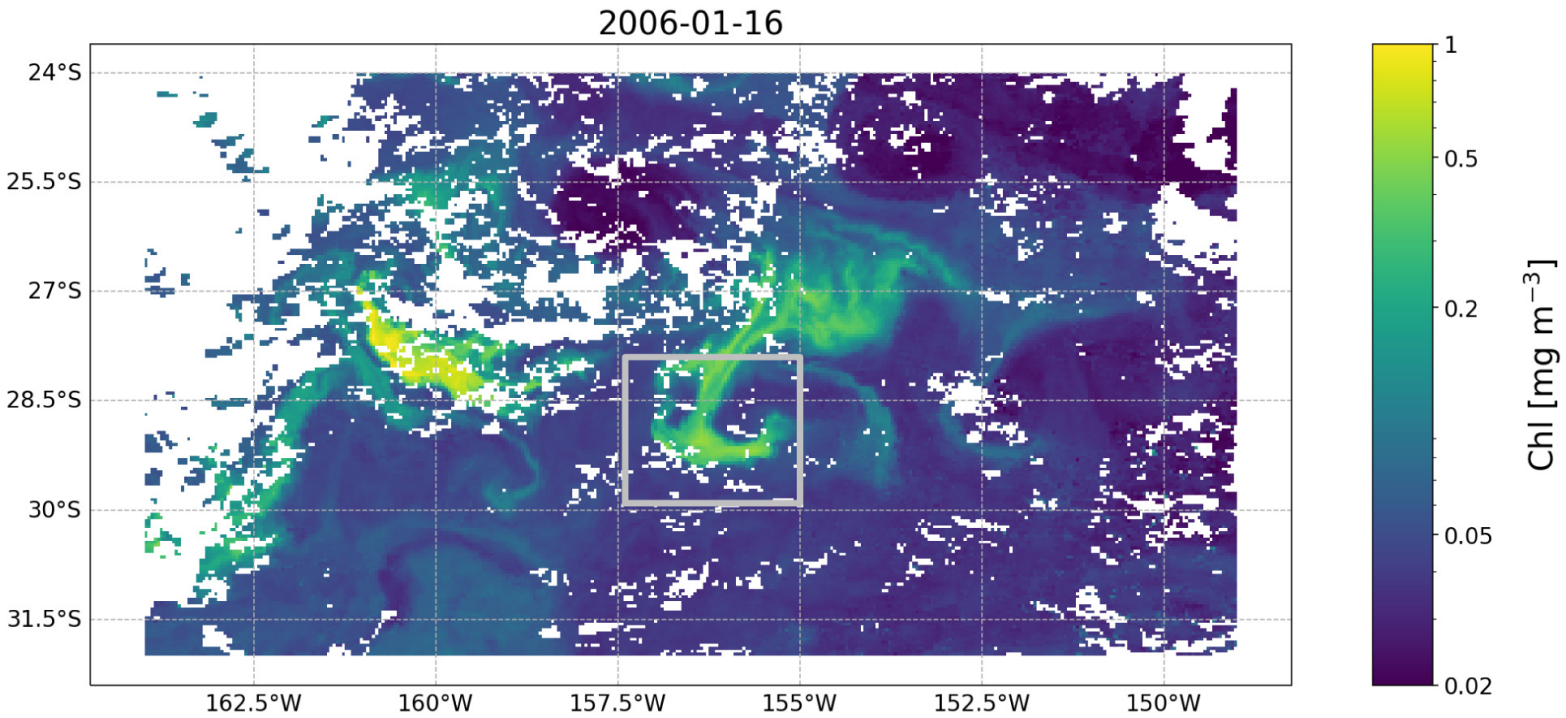
Satellite image of chlorophyll concentration (from the European Space Agency’s Ocean Colour Climate Change Initiative project) taken on 2006-01-16 south east of Madagascar and containing the feature we focus on in Example 3. The feature is the relatively high chlorophyll (i.e., > 0.2 mg m ^−3^) patch within the silver rectangle. White patches are clouds obscuring the image.

**Figure 4.**
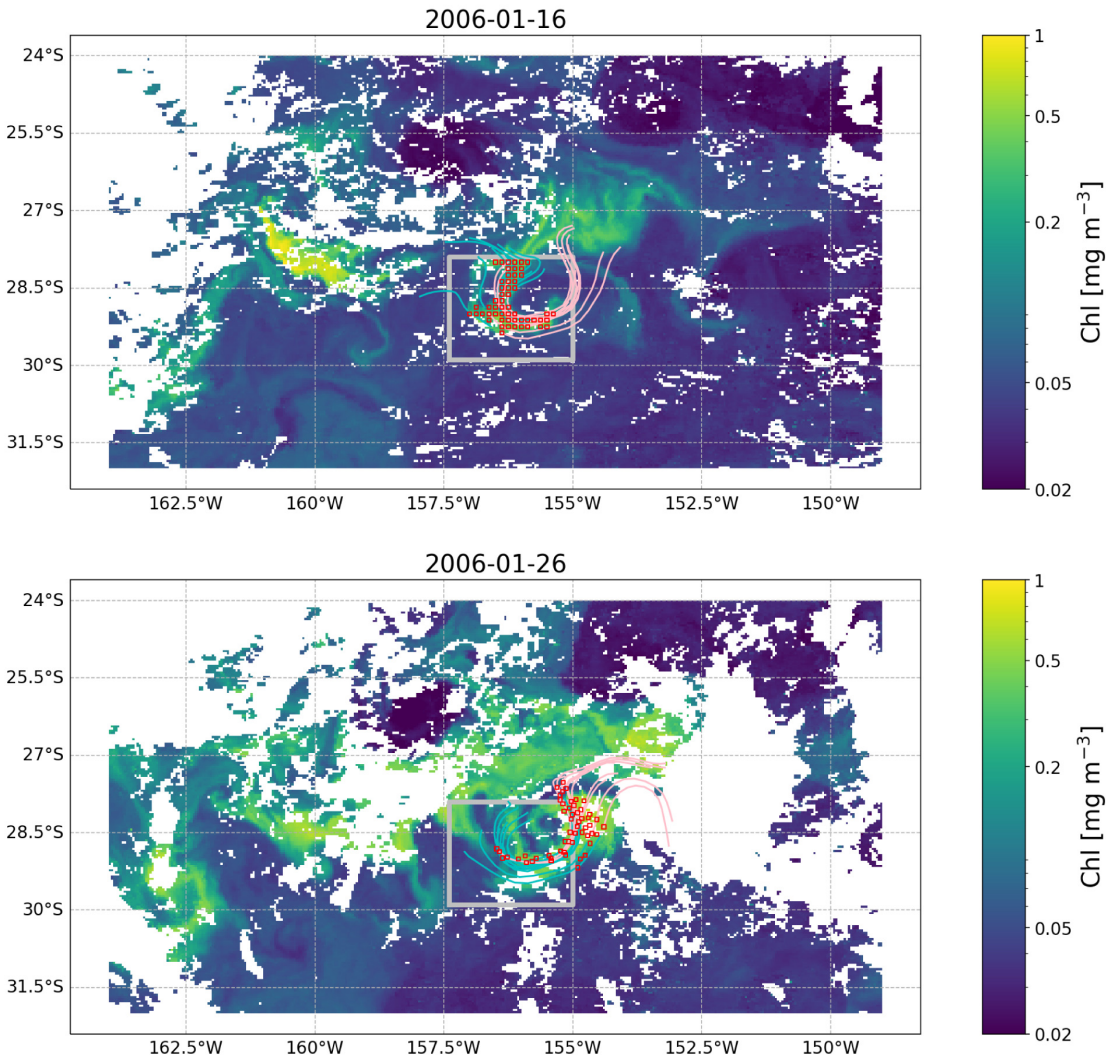
OLTraj product (red squares) matching in time and space the feature observed in the image of Figure 3 on 2006-01-16 (top) and 2006-01-26 (bottom). Some of the backward (cyan lines) and forward (pink lines) Lagrangian trajectories are plotted for each snapshot. Each trajectory extends for 15 days. White patches are clouds obscuring the image.

**Figure 5.**
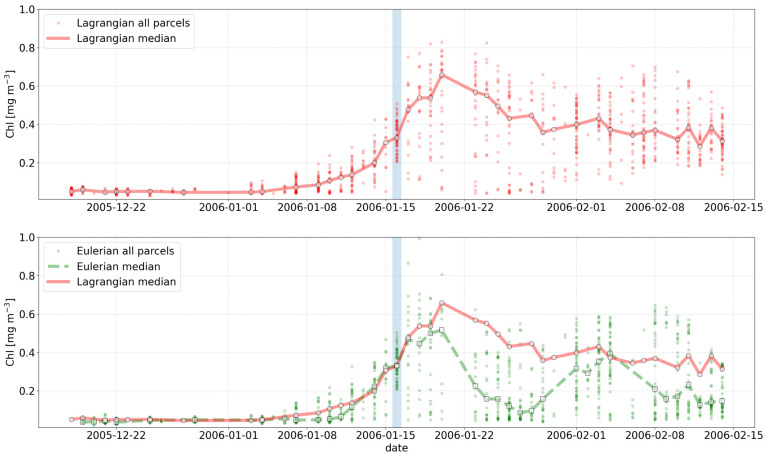
Comparison of chlorophyll concentrations extracted from the Lagrangian (top plot) vs. Eulerian (bottom plot) time series. Small red circles and continuous line represent the chl values extracted along the Lagrangian trajectory of each water parcel and their median (also marked with larger red circles), respectively. Green small circles and dashed line represent the Eulerian time series, i.e., chl values extracted at the location of the initial observation (light blue vertical bar) and their median (also marked with larger green squares), respectively. For both time-series, medians were computed for a given date only if at least 10 of the 54 points were associated with valid chlorophyll observations (i.e. not with cloud pixels). The bottom plot demonstrates the differences in the median Eulerian (green dashed line) and Lagrangian (red continuous line) time series.

***Example 3: Following the evolution of a water mass over time ( Binder Launcher)***. The objective of this example is to demonstrate how the temporal evolution of the properties of a given surface water mass can be tracked in a Lagrangian framework. Specifically, the example focuses on demonstrating how the chlorophyll concentration (chl) measured at a given location evolved before and after the initial observation as the water masses moved. We start by assuming we have observed a chl feature in a given image ( Figure 3). We now want to understand how this feature evolved before and after our initial observation at time 2006-01-15. To do so, we first extract the OLTraj product at that date and then select the starting points ( trajlat and trajlon at time(30)) for the trajectories that overlap with the feature (red squares in Figure 4). We then interpolate the chl data in time and space over the OLTraj Lagrangian trajectories in order to extract the chl values at the locations that the water mass occupied before and after the initial observation. We can finally plot the values of chl along the Lagrangian trajectories ( Figure 5 top) and compare them with the values of chl we would have obtained by extracting time series of chl from the locations of the initial observation (Eulerian time series, Figure 5 bottom). The example also generates an interactive figure where the location of the Lagrangian parcels can be visualised over the corresponding chl image at any date for 29 days before and after the initial observation.

## Dataset validation

### Advantages

**Figure 6.**
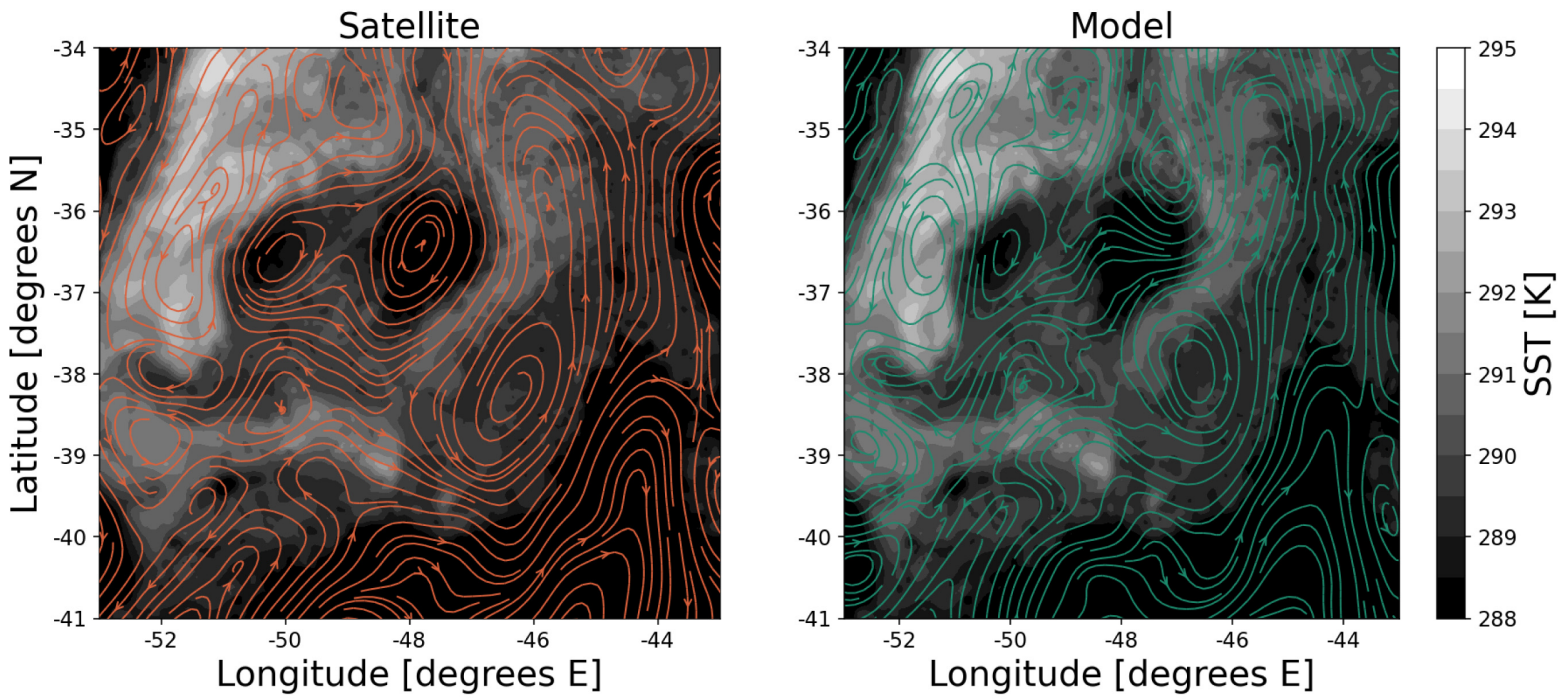
Example of a comparison of satellite-based streamlines from geostrophic currents (left plot, orange lines, from AVISO product) and streamlines from modelled currents (right plot, green lines, from COPERNICUS 1/12 degree reanalysis), superimposed on sea-surface temperature product (grey colours, GHRSST) for the south-west Atlantic on Oct. 4th, 2018. The locations of cold-core eddies (dark grey areas in the centre of the plot) in the centre of the image agree with the AVISO streamlines, but not with the model-reanalysis streamlines.

OLTraj was created to encourage, when needed, non-expert users to interpret in-situ and satellite observations by taking into account that surface water masses move. OLTraj provides pre-computed Lagrangian trajectories, which allow non-experts to skip this computing step and focus on analysing their data in a Lagrangian framework. Furthermore, we provide Python examples to demonstrate how to use OLTraj in practical and common applications. A final but important advantage of OLTraj is that it is based on satellite-derived geostrophic velocities. Thus, OLTraj has better spatio-temporal coherence with other satellite products, such as ocean colour and sea-surface temperature, than modelled velocities (see example in Figure 6).

### Limitations

We do not intend to promote OLTraj as the ultimate tool for Lagrangian analysis. Many other state-of-the-art methods are available ( van Sebille *et al.*, 2018). Instead, the focus of OLTraj is to broaden the application of Lagrangian analysis to non-physical oceanographers. As such, OLTraj has several limitations.

One of the main limitations is inherent in its nature. Being a set of pre-computed trajectories, OLTraj has pre-defined spatial resolution (1/8°) and temporal extent (±29 days). These spatial and temporal characteristics were defined based on a trade off between the number and length of the trajectories computed and the size of the dataset produced that consists of daily files spanning over 22 years (the current overall size is approximately 6.3 Tb). To reduce the burden of storing the entire dataset in a local machine, we have exploited the UK CEDA THREDDS server, which allows users to subset the data before downloading them. Access via the THREDDS server is however necessarily slower than reading files stored on a local machine. We therefore encourage the users to assess if local or THREDDS access is needed for any specific analysis. The overall length of the dataset (1998–2019) was decided based on the extents of i) the satellite ocean-colour record and ii) of the reprocessed gridded geostrophic velocity product when the OLTraj product was computed.

The pre-defined length of the OLTraj product (±29 days) could potentially be seen as another limitation. Yet, methods could be devised to combine multiple OLTraj files and estimate longer Lagrangian trajectories.

OLTraj trajectories are as good as the velocities from which they are derived. Although accurate in the open ocean, the gridded geostrophic velocities used to compute OLTraj have limitations near the coast (i.e., within ~50 km from the shore; e.g., Bouffard *et al.*, 2010; Nencioli *et al.*, 2011; Volkov *et al.*, 2007). These are in part due to technical limitations of satellite altimeters near the coast ( Vignudelli *et al.*, 2019), and in part due to the methodologies used to grid the multi-satellite along-track observations which, although recently improved in the coastal region, are optimized for the open ocean ( Taburet *et al.*, 2019). For these reasons, OLTraj should be used with caution in coastal regions.

Finally, the current version (v2.2) of the OLTraj product is derived from satellite-based geostrophic velocities and therefore only accounts for the mesoscale (i.e. O(100 km)) currents resulting from the balance between the pressure gradient and the Coriolis force ( Isern-Fontanet *et al.*, 2017). Additional non-geostrophic currents are not included in the CMEMS surface velocities and hence nor in OLTraj. These currents are i) currents due to wind stress on the surface ocean (such as Ekman currents and near-inertial oscillations) ( Rio *et al.*, 2014; d'Ovidio *et al.*, 2015)), ii) tidal currents ( Stammer *et al.*, 2014), iii) currents induced by waves (stokes drift), Onink *et al.*, 2019), iv) inertial oscillations ( Elipot *et al.*, 2010), and v) the currents associated with ageostrophic processes typically occurring at scales of O(≤10 km). ( McWilliams *et al.*, 2019).

## Data availability

### Underlying data

The original Copernicus data (January 1998 - December 2019) is available from: https://resources.marine.copernicus.eu/product-detail/SEALEVEL_GLO_PHY_L4_REP_OBSERVATIONS_008_047/INFORMATION

CEDA: Global ocean Lagrangian trajectories based on AVISO velocities, v2.2.

http://doi.org/10.5285/5c2b70d069cb467ab73e80b84c3e395a

This dataset contains the following underlying data:

- Daily files from 1998-01-01 to 2019-12-31.

This dataset is available under the terms of the Creative Commons Attribution 4.0 International license (CC-BY 4.0).

## Software availability

Source code to compute OLTraj available from: https://github.com/grgdll/OLTraj

Archived source code at time of publication: https://doi.org/10.5281/zenodo.5082983

The repository for the examples is: https://github.com/grgdll/OLTraj_examples

Archived source code at time of publication: https://doi.org/10.5281/zenodo.5518531

License: CC BY 4.

## References

[ref-1] Blanke B Speich S Madec G : A global diagnostic of interocean mass transfers. *J Phys Oceanogr.* 2001;31(6):1623–1632. 10.1175/1520-0485(2001)031<1623:AGDOIM>2.0.CO;2

[ref-2] Bouffard J Pascual A Ruiz S : Coastal and mesoscale dynamics characterization using altimetry and gliders: A case study in the Balearic sea. *J Geophys Res Oceans.* 2010;115(C10). 10.1029/2009JC006087

[ref-5] Döös K : Interocean exchange of water masses. *J Geophys Res Oceans.* 1995;100(C7):13499–13514. 10.1029/95JC00337

[ref-25] d'Ovidio F Della Penna A Trull TW : The biogeochemical structuring role of horizontal stirring: Lagrangian perspectives on iron delivery downstream of the Kerguelen Plateau. *Biogeosciences* 2015;12(19):5567–5581. 10.5194/bg-12-5567-2015

[ref-6] d’Ovidio F Fernández V Hernández-García E : Mixing structures in the Mediterranean sea from finite-size lyapunov exponents. *Geophys Res Lett.* 2004;31(17). 10.1029/2004GL020328

[ref-7] Elipot S Lumpkin R Prieto G : Modification of inertial oscillations by the mesoscale eddy field. *J Geophys Res-Oceans.* 2010;115(C9). 10.1029/2009JC005679

[ref-8] Falcini F Corrado R Torri M : Seascape connectivity of European anchovy in the central Mediterranean Sea revealed by weighted Lagrangian backtracking and bio-energetic modelling. *Sci Rep.* 2020;10(1):18630. 10.1038/s41598-020-75680-8 33122692 PMC7596485

[ref-10] Isern-Fontanet J Ballabrera-Poy J Turiel A : Remote sensing of ocean surface currents: A review of what is being observed and what is being assimilated. *Nonlin Processes Geophys.* 2017;24(4):613–643. 10.5194/npg-24-613-2017

[ref-11] Jönsson BF Salisbury JE Mahadevan A : Extending the use and interpretation of ocean satellite data using Lagrangian modelling. *Int J Remote Sens.* 2009;30(13):3331–3341. 10.1080/01431160802558758

[ref-12] Lehahn Y d’Ovidio F Lévy M : Long range transport of a quasi isolated chlorophyll patch by an Agulhas ring. *Geophys Res Lett.* 2011;38(16). 10.1029/2011GL048588

[ref-13] Lumpkin R Johnson GC : Global ocean surface velocities from drifters: Mean, variance, El Niño–Southern Oscillation response, and seasonal cycle. *J Geophys Res Oceans.* 2013;118(6):2992–3006. 10.1002/jgrc.20210

[ref-9] McWilliams JC Gula J Molemaker MJ : The Gulf Stream north wall: Ageostrophic circulation and frontogenesis. *J Phys Oceanogr.* 2019;49(4):893–916. 10.1175/JPO-D-18-0203.1

[ref-14] Nencioli F d'Ovidio F Doglioli AM : Surface coastal circulation patterns by in-situ detection of Lagrangian coherent structures. *Geophys Res Lett.* 2011;38(17). 10.1029/2011GL048815

[ref-15] Nencioli F Dall'Olmo G Quartly GD : Agulhas ring transport efficiency from combined satellite altimetry and argo profiles. *J Geophys Res Oceans.* 2018;123(8):5874–5888. 10.1029/2018JC013909

[ref-17] Onink V Wichmann D Delandmeter P : The role of Ekman currents, geostrophy, and stokes drift in the accumulation of floating microplastic. *J Geophys Res Oceans.* 2019;124(3):1474–1490. 10.1029/2018JC014547 31218155 PMC6559306

[ref-18] Raitsos DE Brewin RJW Zhan P : Sensing coral reef connectivity pathways from space. *Sci Rep.* 2017;7(1):9338. 10.1038/s41598-017-08729-w 28839286 PMC5571014

[ref-19] Rio MH Mulet S Picot N : Beyond GOCE for the ocean circulation estimate: Synergetic use of altimetry, gravimetry, and in situ data provides new insight into geostrophic and Ekman currents. *Geophys Res Lett .* 2014;41(24):8918–8925. 10.1002/2014gl061773

[ref-20] Stammer D Ray RD Andersen OB : Accuracy assessment of global barotropic ocean tide models. *Rev Geophys.* 2014;52(3):243–282. 10.1002/2014RG000450

[ref-21] Taburet G Sanchez-Roman A Ballarotta M : Duacs DT2018: 25 years of reprocessed sea level altimetry products. *Ocean Sci.* 2019;15(5):1207–1224. 10.5194/os-15-1207-2019

[ref-22] van Sebille E Griffies SM Abernathey R : Lagrangian ocean analysis: Fundamentals and practices. *Ocean Model (Oxf).* 2018;121:49–75. 10.1016/j.ocemod.2017.11.008

[ref-23] Vignudelli S Birol F Benveniste J : Satellite altimetry measurements of sea level in the coastal zone. *Surv Geophys.* 2019;40:1319–1349. 10.1007/s10712-019-09569-1

[ref-24] Volkov DL Larnicol G Dorandeu J : Improving the quality of satellite altimetry data over continental shelves. *Geophys Res Lett.* 2007;112(C6). 10.1029/2006JC003765

