# Supplementary material for: Ocean Lagrangian Trajectories (OLTraj): Lagrangian analysis for non-expert users
Source: Open Res Eur. 2021 Nov 15;1:117. Originally published 2021 Sep 30. [Version 2] doi: 10.12688/openreseurope.14133.2 (PMC10445858; doi:10.12688/openreseurope.14133.2)
Supplement: Peer review history

# Peer review: Reviewer response for version 2

**DOI:** 10.21956/openreseurope.15413.r28036

**Published:** 2021-11-17

**Authors:** Alice Della Penna

**Affiliations:** 1 University of Auckland, Auckland, New Zealand

I appreciate the edits that the authors I've put together. I'm very happy to encourage you to accept this Data Note as ready for indexing.

Are sufficient details of methods and materials provided to allow replication by others?

Yes

Is the rationale for creating the dataset(s) clearly described?

Yes

Are the datasets clearly presented in a useable and accessible format?

Yes

Are the protocols appropriate and is the work technically sound?

Yes

Reviewer Expertise:

NA

I confirm that I have read this submission and believe that I have an appropriate level of expertise to confirm that it is of an acceptable scientific standard.

# Peer review: Reviewer response for version 1

**DOI:** 10.21956/openreseurope.15238.r27802

**Published:** 2021-10-27

**Authors:** Joan Llort

**Affiliations:** 1 Institute for Marine and Antarctic Studies, University of Tasmania, Hobart, TAS, Australia

The data note "Ocean Lagrangian Trajectories (OLTraj): Lagrangian analysis for non-expert users" by Dall'Olmo and co-authors provides an easy-to-use toolbox for analysing surface water-mass trajectories in the ocean.

 The note is well written, provides three different and illustrative examples that can be run online thanks to Juptyer Notebooks and MyBinder and clearly details all the data sources and limitations of the approach. The goal of this tool is to allow the general community on oceanography to incorporate Lagrangian trajectories analysis in observations works. As stated in the introduction of the note, Lagrangian analyses are of great help to interpret ocean observations, particularly for studies focusing on phytoplankton and ocean biogeochemistry. These methods have been used several times in the past for a number of cruise surveys but so far, they required at least one or two experts in physical oceanography and satellite data to implement them. OLTraj breaks this barrier by simplifying the implementation of the approach so any researcher with minimal programming skills can apply it to its own zone/period of interest.

 I strongly support the indexing of this note in its current version.

Are sufficient details of methods and materials provided to allow replication by others?

Yes

Is the rationale for creating the dataset(s) clearly described?

Yes

Are the datasets clearly presented in a useable and accessible format?

Yes

Are the protocols appropriate and is the work technically sound?

Yes

Reviewer Expertise:

Ocean biogeochemistry, Physical oceanography, Observations, (sub)mescoscale circulation

I confirm that I have read this submission and believe that I have an appropriate level of expertise to confirm that it is of an acceptable scientific standard.

# Response

**Published:** 2021-11-03

**Authors:** Giorgio Dall'Olmo

We thanks Dr Llort for their positive review.

# Peer review: Reviewer response for version 1

**DOI:** 10.21956/openreseurope.15238.r27732

**Published:** 2021-10-15

**Authors:** Alice Della Penna

**Affiliations:** 1 University of Auckland, Auckland, New Zealand

This *Data Note* provides good information for an exciting dataset that is aimed at encouraging non-specialists to work with Lagrangian re-analyses of satellite altimetry. I think tools like this are very valuable for interdisciplinary research and I also think this could be a good tool for teaching – exposing students to these approaches early in their studies. Overall, this is a very positive review. I only have a few small comments that I think could be helpful.

1. Most of the potential users of this dataset are likely to have limited experience in Python so I think it’s great that Python notebooks for the examples are made available. My suggestion is to include the links to each notebook individually in the text and not only at the end of the manuscript (and in the heading paragraph of the examples section) so that a reader can potentially look at them in parallel while reading the text. So, for example, just after “ *Example 1: Plotting trajectories around a fixed-point station” *I would recommend including the link to the notebook – even if is already mentioned at the end of the manuscript.
2. In the “Advantages” section, I do understand the point the authors are making about how using altimetry-based currents makes matches with biogeochemical data more accurate compared to models. And I totally agree. However, I’m not sure that many readers who are not familiar with this kind of analyses would understand exactly what that means. Would there be any reference with an example of a mismatch the authors could include? Or could they add a figure showing an example where a model shows a mismatch but not the altimetry? Perhaps just a schematic showing a possible scenario?
3. In the “Limitations” section, I think the authors do a good job at describing the ageostrophic components. I recommend adding some references for readers who might want to read more about each different component and mention that previous studies that have incorporated estimates of ageostrophic components have increased the accuracy of the near-surface currents (e.g. d’Ovidio *et al*., 2015).

Are sufficient details of methods and materials provided to allow replication by others?

Yes

Is the rationale for creating the dataset(s) clearly described?

Yes

Are the datasets clearly presented in a useable and accessible format?

Yes

Are the protocols appropriate and is the work technically sound?

Yes

Reviewer Expertise:

My area of expertise encompasses the use of Lagrangian re-analyses. In particular, I have been using tools such as the ones described in this Data Note to interpret oceanographic observations, understand the relationship between tracked animals and their environment, and designed tools to aid the spatial planning for Marine Protected Areas.

I confirm that I have read this submission and believe that I have an appropriate level of expertise to confirm that it is of an acceptable scientific standard, however I have significant reservations, as outlined above.

## References

[1] : The biogeochemical structuring role of horizontal stirring: Lagrangian perspectives on iron delivery downstream of the Kerguelen Plateau. *Biogeosciences* .2015;12(19) : 10.5194/bg-12-5567-2015 5567-5581 10.5194/bg-12-5567-2015

# Response

**Published:** 2021-11-03

**Authors:** Giorgio Dall'Olmo

We thanks Dr Della Penna for their positive and constructive review. In the revised version of the manuscript we have addressed all suggestions, which we believe have improved the manuscript.
